# Sustainable Fishmeal Alternatives: Impact of Partially Defatted Black Soldier Fly (*Hermetia illucens*) Meal on Growth and Health of Yellowtail Kingfish (*Seriola lalandi*)

**DOI:** 10.1155/anu/1804215

**Published:** 2025-06-19

**Authors:** Luke Pilmer, Lindsey Woolley, Alan Lymbery, Michael Salini, Chinh Dam, Md Javed Foysal, Gavin Partridge

**Affiliations:** ^1^Centre for Sustainable Aquatic Ecosystems, Harry Butler Institute, Murdoch University, Murdoch 6150, Western Australia, Australia; ^2^Department of Primary Industries and Regional Development, Fremantle 6160, Western Australia, Australia; ^3^Ridley Aquafeed Pty Ltd., Narangba 4504, Queensland, Australia; ^4^Nutrition and Seafood Laboratory (NuSea.Lab), School of Life and Environmental Sciences, Deakin University, Queenscliff, Victoria, Australia; ^5^Research Institute for Aquaculture No.1, Dinh Bang, Tu Son, Bac Ninh, Vietnam; ^6^School of Environmental and Life Sciences, University of Newcastle, Callaghan 2308, New South Wales, Australia; ^7^Oceans Institute, University of Western Australia, Crawley 6009, Western Australia, Australia

**Keywords:** fishmeal replacement, insect protein, microbiome, sustainable aquafeeds, yellowtail kingfish

## Abstract

Reducing fishmeal (FM) in aquaculture diets is essential for improving sustainability and reducing reliance on marine resources. Black soldier fly (BSF; *Hermetia illucens*) larvae meal is a promising alternative protein source. This study evaluated the effects of replacing FM with BSF meal on the growth and health of juvenile yellowtail kingfish (YTK) (*Seriola lalandi*, initial weight ~22 g). Fish were reared in 24 tanks (three replicates per treatment) and fed for 33 days under controlled conditions. Eight diets were tested: a control (40% FM) and seven diets with BSF meal replacing FM at 25%, 50%, or 75%, with or without garlic and tuna hydrolysate additives. Fish fed 25% and 50% BSF diets showed growth and feed conversion comparable to the control, while 75% BSF significantly reduced growth due to decreased feed intake. Additives did not improve feed intake. Histological analysis indicated good gut health and nutrient absorption. Serum cholesterol decreased with BSF inclusion, and urea levels varied. No significant changes in gene expression were observed in the gut, liver, or brain. Microbiome analysis showed increased diversity and compositional shifts at higher BSF levels. These results support the use of BSF meal as a sustainable FM replacement at moderate inclusion levels, with further research needed to address palatability at higher levels.

## 1. Introduction

The limited availability of fishmeal (FM) continues to threaten the economic and environmental sustainability of aquaculture. Global FM production has remained relatively stable over the past two decades, fluctuating between 4.5 and 5 million tonnes annually, influenced by factors such as climate variability and regulatory measures. Notably, in 2022, ~17 million tonnes of aquatic animal production were directed towards non-food uses, primarily to produce FM and fish oil, accounting for 83% of non-food utilisation [1]. Furthermore, the reliance on FM and fish oil derived from wild fish stocks for aquafeeds contributes significantly to the environmental pressures associated with aquaculture. This dependency can lead to overfishing, depletion of wild fish populations, and disruption of marine ecosystems, ultimately threatening biodiversity. Additionally, the extraction and processing of FM and fish oil result in a substantial carbon footprint, further exacerbating greenhouse gas emissions [2]. To mitigate these environmental impacts, it is essential to reduce dependence on these finite resources by incorporating more sustainable alternatives, which is critical for ensuring the ecological sustainability of aquaculture systems over the long term [3]. Advancements in this area are vital for maintaining the viability of intensive fish farming as a sustainable source of protein for human diets. As a result, there has been significant research into identifying sustainable and nutritionally adequate alternatives to FM and fish oil. Promising candidates include terrestrial plant proteins, animal by-products, microalgae, macroalgae, bacteria, and insects [4–8]. Among these, insect-based ingredients have garnered increasing attention as potential feed components for terrestrial and aquatic species, reflected in the growing body of scientific literature on the topic [4, 5, 9–12].

The larvae of the black soldier fly (BSF: *Hermetia illucens*) are considered an important candidate for animal feeds due to their high nutritional value, rapid growth rate, and ability to convert organic waste into high-quality protein and fat. Previous studies have shown that BSF larvae can effectively replace traditional feed ingredients, such as FM and soybean meal, reducing the environmental impact of feed production [13–15]. Furthermore, their use in animal feeds has been associated with improved growth performance and health benefits in various livestock and aquaculture species [10, 16]. On a dry matter (DM) basis, BSF larval meal typically contains 37%–80% protein, depending on rearing conditions and processing, with a well-balanced essential amino acid (AA) profile. The fat content is ~35%, which can be reduced to 5%–9% through defatting [17–19]. Defatting improves the suitability of BSF meal for aquafeeds by increasing protein concentration and reducing excess dietary lipids, which can otherwise impair feed intake and growth in marine species. A defatted BSF meal contains significantly more crude protein and less fat than a full-fat meal, making it more appropriate for aquaculture [15]. In several species, defatting has shown clear nutritional advantages, it improved lipid metabolism and reduced fat accumulation in Japanese seabass without compromising growth [20] and supported gut health and performance in turbot [21]. Combined with its high digestibility, these improvements position BSF meal as a promising and adaptable alternative protein source for aquaculture feeds.

Building on its improved nutritional profile, BSF meal has primarily been evaluated as a feed ingredient for freshwater fish species such as tilapia (*Oreochromis niloticus*) [22–24], rainbow trout (*Oncorhynchus mykiss*) [25, 26], and African catfish (*Clarias gariepinus*) [27], showing promising results in terms of growth and feed utilisation. However, interest in its application in marine aquaculture is growing, with studies conducted on marine fish such as turbot (*Scophthalmus maximus*) [28] and European seabass (*Dicentrarchus labrax*) [19]. Belghit et al. [5] also demonstrated that BSF meal can effectively replace FM in diets for sea-water phase Atlantic salmon (*Salmo salar*) without negatively affecting growth performance, feed utilisation, nutrient digestibility, liver health, or fillet sensory qualities. Despite this, concerns have been raised regarding the palatability of BSF-based diets, as some fish species exhibit reduced feed intake when BSF meal is included at higher levels [28]. Studies investigating the use of BSF meal in large pelagic carnivorous finfish species such as yellowtail kingfish (YTK, *Seriola lalandi*) are still lacking.

To overcome potential issues with palatability, various dietary additives, including garlic (*Allium sativum*) and fish protein hydrolysates, have been studied as potential enhancers. Garlic has been reported to improve feed palatability, support growth, and enhance bacterial resistance in several fish species [29–32]. Likewise, the addition of fish protein hydrolysates to aquafeeds has been associated with improved growth, immune response, and palatability [33–35]. Therefore, this study combined garlic and tuna hydrolysate with BSF meal to investigate potential synergistic benefits in YTK diets. This experiment specifically assessed the effect of replacing FM with a BSF meal on the growth and health of YTK, both with and without the inclusion of these palatability enhancers.

## 2. Methods

### 2.1. Diet Formulation

The BSF meal used in this study was obtained from Future Green Solutions (Perth, Western Australia). The larvae were reared on a diet consisting of vegetable and grain waste and harvested at the larval stage, prior to pupation, to minimise chitin accumulation. The meal underwent partial defatting, with proteins separated at 90°C, followed by air drying for 6 h.  Table 1 presents the proximate composition and AA profile of the BSF meal in comparison to FM. Four isonitrogenous (crude protein: 51%) and isolipidic (crude fat: 18%) diets were formulated to include BSF meal at inclusion levels of 0%, 10%, 20%, and 30%, corresponding to 0%, 25%, 50%, and 75% replacement of FM (Table 2). The control diet, which contained no BSF meal, was based on the formulation described by Pilmer et al. [36]. All diets were designed to meet the essential nutritional requirements for optimal juvenile YTK growth and performance [36, 37].

Each of the four diets was also prepared in variations that included additives of liquid tuna hydrolysate (69% water; SAMPI, Australia) and garlic powder (Spencers, Australia) at inclusion levels of 30 g/kg and 10 g/kg DM, respectively. Yttrium oxide was incorporated into all diets as an indigestible marker for digestibility analyses. The diets were processed into 5 mm pellets using a cold-press extruder (Specialty Feeds, Glen Forrest, Western Australia).

### 2.2. Feeding Trial and Growth Monitoring

The YTK used in this experiment were derived from naturally spawned eggs produced by captive broodstock housed at DPIRD's Fremantle Marine Fish Hatchery (Perth, Western Australia). The broodstock was maintained in indoor 10,000 L tanks with a continuous flow of seawater at a temperature of 20°C. Prior to the trial, 360 fish with an average weight of 58.7 ± 0.2 g were individually weighed, microchipped, and distributed among 24 450 L tanks, with each treatment replicated three times and 15 fish per tank. The tanks were supplied with oxygenated seawater at ambient temperature (20°C ± 1°C) at a flow rate of 6 L/min in a flow-through system.

The fish were fed the experimental diets twice daily to apparent satiety over a 33-day period, and feed intake was recorded daily. At the conclusion of the growth trial, individual body weights were measured for all fish, and performance metrics such as weight gain, feed conversion ratio (FCR), and specific growth rate (SGR) were calculated using the following formulae [38, 39]:

  $$\begin{array}{c} \text{Weight gain }\left(g.{\text{fish}}^{-1}\right)=\text{ final }w\text{eight}-\text{initial }w\text{eight}, \end{array}$$

  $$\begin{array}{c} \text{Specific growth rate }\left(\text{SGR},\text{ \% BW }{\text{day}}^{-1}\right)=\frac{\left(\ln\text{final }w\text{eight}-\ln\text{initial }w\text{eight}\right)}{\text{days}}\times 100, \end{array}$$

  $$\begin{array}{c} \text{Feed conversion ratio }\left(\text{FCR}\right)=\frac{\text{feed consumed}}{\text{weight gain}}. \end{array}$$

The experimental fish were fasted for 24 h prior to commencing sampling. Three fish per tank were sampled at the end of the growth trial, with blood and tissue samples collected for analyses of biochemical parameters, gut morphology and function, gene expression, and microbiome composition.

### 2.3. Biochemical Parameters

Blood samples were collected from the caudal vein and transferred into 2 mL Eppendorf tubes. The samples were left to clot at room temperature (~20°C) for 1 h before being stored at 4°C overnight. Following this, the samples were centrifuged at 3000 *g* for 10 min at 4°C. The resulting serum was carefully separated, divided into triplicate aliquots of 200 μL, and frozen for subsequent analysis. Serum parameters, including cholesterol, alanine aminotransferase (ALT), glutamate dehydrogenase (GLDH), lipase, urea, total protein, and triglycerides, were measured using a Beckman Coulter chemical analyser (AU 680, USA).

### 2.4. Gut Morphology and Function

Liver and hindgut samples were collected and preserved in 10% neutral buffered formalin for histological analysis. Tissue sections were stained using haematoxylin and eosin (H&E) and PAS-Alcian Blue. Hindgut sections were evaluated to determine the lamina propria area as a percentage of the villus area (% lamina propria) and the thickness of the muscularis layer. Additional hindgut samples were immediately frozen in liquid nitrogen for myeloperoxidase (MPO) analysis, which served as a marker for neutrophil infiltration associated with enteritis [40].

The method for MPO activity used was the same as described in Woolley et al. [41]. Briefly, the hindgut samples were snap-frozen in liquid nitrogen. Tissue was ground using a pre-chilled mortar and pestle and homogenised in 0.5% hexadecyltrimethylammonium bromide in 50 mM PBS, following the method described by Woolley et al. [41]. The homogenate was centrifuged, and the supernatant was diluted 1:1500 in the same buffer. Samples, in triplicate, were then assayed in a 384-well microplate using a fluorescence-based method with 10 µM APF and 10 µM hydrogen peroxide (excitation at 485 nm; emission 515–530 nm). MPO activity was quantified against human MPO standards and normalised to protein content determined via a Bio-Rad protein assay at 750 nm.

### 2.5. Gene Expression

Hindgut, liver, and brain samples were taken from control fish and those from the highest and lowest level of FM replacement (BSF 25 and 75) with and without additives for analysis of gene expression relating to digestion, growth, and immune function (Table 3). These samples were placed in a tube with RNAlater (1.5 mL; Ambion), stored at 4°C for 24 h. and then transferred to a −80°C freezer until extraction. Further details on the genes tested, specific primers used for qPCR, and sampling details can be found in Tables S1 and S2.

Total RNA was extracted from 30 mg of tissue preserved in RNAlater using the RNeasy Mini Kit (Qiagen, Australia). Tissue samples were disrupted and homogenised in 600 µL of buffer RLT with a TissueRuptor (Polytron, USA). To facilitate RNA binding to the RNeasy membrane, 70% ethanol was added to the lysate. The mixture was then transferred to a RNeasy mini spin column fitted in a 2 mL collection tube. The RNA extraction process included an initial wash with buffer RW1 for 15 s (≥10,000 rpm), followed by two washes with buffer RPE for 2 min and 15 s. The final pellet was dissolved in 30–50 µL of RNase-free water (Qiagen, Australia).

The RNA concentration was quantified using a Nanodrop spectrophotometer (ND-2000), and RNA integrity was evaluated with the Agilent 2100 Bioanalyzer (Agilent Technologies, CA, USA) using RNA Nano Chips and the RNA 6000n Assay Kit (Agilent Technologies, CA, USA). Extracted RNA samples were stored at −80°C for subsequent analysis.

Gene expression levels were quantified by RT-qPCR and normalised against three reference genes: β-actin, elongation factor 1-alpha (ef1α), and 18S rRNA, which were selected based on their stable expression across experimental treatments and tissues.

### 2.6. Microbiome

The hindgut was carefully dissected under aseptic conditions using sterile instruments, and the samples were promptly transferred into sterile tubes containing RNAlater to ensure the preservation of microbial DNA integrity before further analysis. Microbial DNA extraction from the hindgut of the experimental fish was performed utilising the QIAamp 96 DNA QIACube HT kit (Cat# 51331, Qiagen, Germany) in following with the provided protocol. Approximately 20 mg of whole tissue was subjected to digestion with proteinase at 56°C for 1 h, followed by DNA extraction. The concentration of the isolated DNA was measured using the Qubit dsDNA High Sensitivity assay (cat #Q32851, ThermoFisher Scientific) and then stored at −20°C for later use. Before constructing the library, DNA samples were adjusted to 10 ng·μL^−1^ concentration and re-verified with the Qubit dsDNA HS assay.

Quantitative sequencing libraries were prepared from tissue samples using Lexogen's QuantSeq 3′ mRNA-Seq Kit (Lexogen, Austria). This transcriptomic method was used separately to assess host gene expression and is not related to the DNA-based microbiome profiling described below. mRNA was selectively captured from total RNA using oligo dT primers. Following the synthesis of the first strand, the RNA template was removed, and second-strand synthesis was initiated using random primers with an Illumina-compatible linker sequence, along with a DNA polymerase. The resulting products were purified through a magnetic bead-based purification process and subsequently amplified by PCR to generate pre-sequencing cDNA libraries. These libraries were sequenced with a read length of 100 bp on an Illumina HiSeq 2500 platform at the Australian Genome Research Facility (AGRF, Melbourne, Australia).

In contrast, microbial community composition was assessed using DNA-based 16S rRNA gene amplicon sequencing targeting the V3–V4 hypervariable regions, also sequenced at AGRF. Raw 16S rRNA paired-end amplicon sequence data were processed using qiime2 software (v2021.11). Quality trimming and amplicon sequence variant (ASV) picking were performed with the DADA2-plugin in qiime2 [42, 43]. The trimming process involved denoising, dereplicating, removing low-quality reads, and filtering chimeric sequences. The paired-end trimming parameters were set to trim 10 bases from the forward and reverse reads and truncate forward and reverse reads at 280 and 260 bases, respectively. ASV tables were further filtered to remove low abundance features (singletons) at a non-zero frequency threshold (—p-min-frequency 10). Multiple sequence alignment was conducted using Mafft [44], and a phylogenetic tree was constructed with Fasttree [45]. Taxonomic classification of representative ASVs into different taxa levels was performed using a pre-trained naive Bayes classifier. The consensus blast method classified the ASV table at a 99% similarity threshold against the SILVA 138 release [46]. Taxonomic data were merged with the feature table based on curated ASVs using the qiime2 function “feature-table-collapse.”

### 2.7. Protein and Energy Deposition

Following the conclusion of the growth trial, the three sampled fish per tank were pooled homogenised, frozen, freeze-dried, and analysed for proximate composition (protein, fat, ash, and energy). Protein, fat, and energy retention were calculated using the following equation:

  $$\begin{array}{c} \text{Retention efficiency of protein},\text{fat and energy}\left(\text{\% }\right)=100\times \left[\frac{\left(\text{final whole body nutrient-initial whole body nutrient}\right)}{\text{total nutrient consumed}}\right]. \end{array}$$

### 2.8. Digestibility Assessment

Following the growth trial, faecal samples were collected from a separate group of fish housed in the same experimental tanks and maintained under identical conditions. Each of the 24 tanks was stocked with 10 fish (average weight: 710 ± 62 g). The fish were allowed a 1-week acclimation period on the experimental diets before the first faecal stripping event, conducted using methods similar to those described by Booth and Pirozzi [47]. Fish were anaesthetised using benzocaine, and the ventral surface was cleaned before gently applying pressure to the abdomen to expel urinary products. The area was cleaned again, and faecal material was extracted from the distal intestine into a sterile 50 mL container using gentle abdominal pressure. The collected faecal samples were immediately stored at −20°C. After the stripping procedure, fish were returned to their respective tanks for recovery.

Faecal collection occurred ~6 h after the last feeding [48]. Each fish was stripped twice over a 5-day period to obtain ~40 g of faeces per tank, with faecal material pooled from all 10 fish per tank. Each treatment had three replicate tanks. The pooled faecal samples were freeze-dried and ground for analysis. The samples were assessed for protein content, gross energy, and yttrium oxide, and apparent digestibility coefficients (ADCs) were calculated using the following equation:

  $$\begin{array}{c} \text{Apparent digestibility coefficient }\left(\text{ADC}\right)\text{of protein},\text{energyand dry matter }\left(\text{\% }\right)=100\times \left[1-\left(\frac{\text{dietary yttrium}}{\text{faecal yttrium}}\right)\times \left(\frac{\text{faecal nutrient}}{\text{dietary nutrient}}\right)\right]. \end{array}$$

### 2.9. Chemical Analysis

DM, crude protein, total lipids, and ash were measured for experimental diets, and whole-body samples. Faecal samples underwent analysis for gross energy using bomb calorimetry, along with measurements of total nitrogen and DM content. The concentration of yttrium in both the diets and faeces was assessed via mixed acid digestion followed by inductively coupled plasma mass spectrometry (ICP-MS). Carbohydrate content was determined by difference, using the formula for nitrogen-free extracts (NFEs): NFE = 100 – crude protein – crude lipid – ash. DM was determined through gravimetric analysis by drying samples in an oven at 105°C for 24 h. Nitrogen content, measured with a Leco auto-analyser, was multiplied by 6.25 to estimate crude protein. Lipid content was extracted using chloroform and methanol (2:1 v/v) following the method of Folch et al. [49] and quantified gravimetrically. Ash content was obtained through combustion in a muffle furnace at 550°C for 12 h. AA profiles of the raw materials and diets were identified using high-performance liquid chromatography (HPLC) after acid hydrolysis, prepared based on the Waters Corp. (1996) protocol for complex feed samples. Post-hydrolysis, AAs were derivatised with the Waters AccQTag Ultra system and analysed on a Waters Acquity Arc UHPLC with photodiode array detection (Waters Corporation, Milford, MA).

### 2.10. Data Analysis

Two-way ANOVA was used to determine the effect of BSF replacement level and inclusion of dietary additives on growth, feed intake, and FCR, as well as biochemical parameters, gut morphology measurements, and gene expression. Data were normalised by arcsine transformation where needed. When significant differences were identified, a post hoc Tukey's test was performed to determine where these differences occurred. To account for the risk of type I errors due to multiple comparisons, the Benjamini–Hochberg procedure was employed to control the false discovery rate at a significance threshold of *p* < 0.05. All statistical analyses were performed using JMP software (version 14, SAS Institute Inc., Lane Cove, Australia).

Microbial alpha diversity was described by observed ASV species richness and the Shannon–Weiner index, and beta diversity (differences in microbial community composition) by unweighted and weighted UniFrac distance metrics. The effects of BSF replacement level and inclusion of dietary additives on alpha diversity measurements were assessed using two-way ANOVA and Tukey's HSD tests. Permutational analysis of variance (PERMANOVA; 1000 permutations) was applied to distance matrices to test the effects of BSF replacement and additives on beta diversity. Beta diversity analyses were conducted using the phyloseq, ape, and vegan (Adonis) R packages. A Venn diagram contrasting shared and unique ASVs was generated using the MicroEco R package [50]. Only taxa with more than 1% read abundance were considered for compositional analysis using the phyloseq R package. Read counts were transformed into percentages to generate relative abundance (RA) plots at the phylum and genus levels. A linear discriminant analysis (LDA) score of 2.0 or higher was used as the threshold to identify differentially abundant taxa, following a Kruskal–Wallis test to detect significant differences in taxa abundance across groups. A Bonferroni adjustment was applied to control for multiple comparisons, with a *p* < 0.05, indicating statistical significance for differential abundance (DA) analysis. Differentially abundant bacteria in various BSF replacement diets, with or without dietary additives, were identified using the MicrobiomeMarker R package [51].

## 3. Results

### 3.1. Proximate Composition

The results of the dietary analyses are found in Table 4. Although the diets were designed to be isonitrogenous, the analysis found a slight increase in crude protein in the BSF50 + diet (52.1%) and a slight reduction in the BSF0 + diet (49.3%). The BSF0 + diet had a higher fat content (21.7%) compared to all other diets (17.3%). Furthermore, dietary cholesterol levels decreased as FM was replaced with BSF meal, with the BSF75 diet having the lowest cholesterol level (1.1%). The AA profile analysis confirmed that the target AA levels were met for each diet [36].

### 3.2. Growth, Feed Intake, and FCR

**Figure 1:**
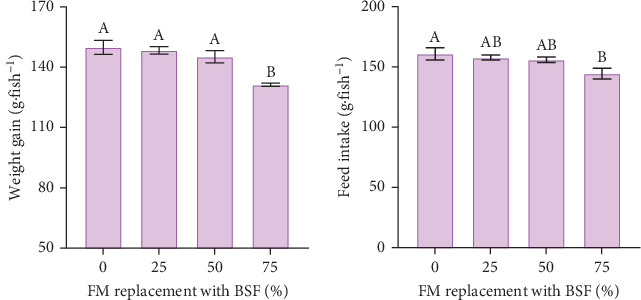
(A) weight gain (g.fish^−1^) and (B) feed intake (g.fish^−1^) of yellowtail kingfish-fed diets containing various levels of fishmeal replacement with BSF. Values are the least-square means ± SE (*n* = 6). Post hoc analysis comparing BSF treatment diets (Tukey *p* < 0.05). Uppercase letters above each bar indicate significant differences between treatments.

Survival during the growth trial was 100% in all treatments, and a summary of the various performance metrics for the eight different diets is given in Table 5. BSF meal replacement level significantly affected weight gain (*F*_3_ = 11.651, *p* < 0.001), with fish fed the BSF75 diet gaining less weight (131.3 ± 1.1 g.fish^−1^) than those fish fed the other diets (pooled average 147.8 ± 3.6 g.fish^−1^) (Figure 1A). There was no significant impact of dietary additives (*F*_1_ = 0.674, *p* = 0.424) or the interaction of BSF replacement level and additives (*F*_3_ = 1.496, *p* = 0.253) on weight gain. Feed intake was significantly affected by BSF inclusion level (*F*_3_ = 4.610, *p* = 0.017), with fish fed the highest BSF inclusion diet consuming significantly less compared to the other diets (Figure 1B), however feed intake was not influenced by dietary additives (*F*_1_ = 2.079, *p* = 0.169) or the interaction of these terms (*F*_3_ = 2.090, *p* = 0.142). Despite the significant reduction in feed intake and weight gain at the highest BSF inclusion level, there was no significant impact of BSF inclusion on FCR (*F*_3_ = 0.360, *p* = 0.783). There was also no significant impact of dietary additives (*F*_1_ = 0.363, *p* = 0.555) or interaction of BSF replacement and additive inclusion (*F*_3_ = 0.345, *p* = 0.793) on FCR.

### 3.3. Digestibility and Protein and Energy Retention

Final whole-body proximate composition, ADCs, and retention efficiencies are shown in Table 6. The protein content of the whole fish was significantly affected by BSF replacement level (*F*_3_ = 3.7, *p* = 0.033), being lower in the fish fed the BSF50 diet (LSM 64.5% ± 0.6%) compared to the fish fed the BSF75 diet (66.8% ± 0.6%). There was also a significant effect of additives on protein content in whole fish (*F*_1_ = 10.5, *p* = 0.005), with the fish fed the additives having significantly higher protein content (LSM 66.3% ± 0.4%) than the fish fed without additives (LSM 64.8% ± 0.4%). Fat content was not affected by dietary additives but was significantly affected by BSF replacement level, with fish fed the BSF0 diet having significantly lower total body fat content (21.3% ± 0.9%) than the fish fed the BSF50 diets (24.7% ± 0.9%) (*F*_3_ = 4.9, *p* = 0.013). There were no significant effects of either BSF replacement level (*F*_3_ = 2.58, *p* = 0.089) or additives (*F*_1_ = 0.21, *p* = 0.652) on ash content and no significant interactions (*F*_3_ = 0.62, *p* = 0.611) between BSF replacement and additives for any proximate composition parameters.

Due to a lack of significant effects in growth parameters, ADCs were only calculated for the diets without additives. The BSF replacement level did not significantly affect the digestibility of DM (pooled average 83.4% ± 0.8%) (*F*_3_ = 0.9, *p* = 0.479), protein (pooled average 79.4% ± 1.1%) (*F*_3_ = 0.1, *p* = 0.929) or gross energy (pooled average 76.2% ± 0.9%) (*F*_3_ = 0.6, *p* = 0.618) (Table 6).

Protein retention was not significantly affected by BSF replacement level (*F*_3_ = 0.52, *p* = 0.674), dietary additives (*F*_1_ = 3.13, *p* = 0.095) or their interaction (*F*_3_ = 1.66, *p* = 0.215). There was a significant effect of the BSF replacement level on fat retention (*F*_3_ = 16.2, *p* < 0.001), with fish fed the BSF0 diet having significantly lower fat retention (LSM 32.9% ± 1.9%) than fish fed the BSF replacement diets (LSM 42.8% ± 0.7%). Among the BSF replacement diets, there was a significant difference between the BSF50 (46.0% ± 1.9%) and BSF75 diets (40.3% ± 1.9%), with BSF50 showing higher fat retention efficiency. There was also a significant effect of additives on fat retention (*F*_1_ = 7.1, *p* = 0.017), with fish-fed additives having significantly lower fat retention (38.5% ± 1.3%) than those fed diets without additives (42.1% ± 1.3%) (Table 6). There was no significant interaction between BSF replacement level and additives on fat retention (*F*_3_ = 2.56, *p* = 0.091). Energy retention efficiency was significantly influenced by the replacement of FM with BSF (*F*_3_ = 4.8, *p* = 0.014) with an increase in energy retention in those fish fed the BSF50 (LSM 31.8% ± 0.8%) diet compared to the BSF0 (LSM 28.7% ± 0.8%) diets. There was no significant effect of additive (*F*_1_ = 1.96, *p* = 0.180) nor an interaction (*F*_3_ = 0.68, *p* = 0.517) of additive and BSF replacement level on energy retention.

### 3.4. Histological Assessment

The liver and intestinal tissues across all dietary treatments presented similar characteristics, indicating no significant differences among the fish. The livers consistently exhibited adequate glycogen reserves with lipid dispersed in small droplets rather than large vacuoles, suggesting a healthy metabolic state. The hindgut tissues featured long villi with large enterocytes, a notable presence of mucous cells, and numerous absorptive droplets within the hindgut enterocyte cytoplasm. Additionally, there was minimal infiltration of extra cells, such as lymphocytes, and rare occurrences of cell shedding by diapedesis. The lamina propria remained thin across samples, reflecting consistent and healthy gut morphology among the fish.

**Figure 2:**
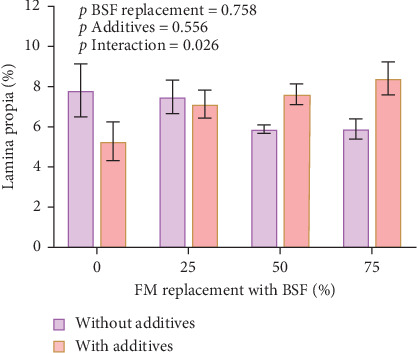
Lamina propria ratio to villi length measured in yellowtail kingfish hindgut following a 33-day growth trial ± SE.

While there were no significant effects of BSF replacement level (*F*_3_ = 0.39, *p* = 0.758) or additive inclusion (*F*_1_ = 0.36, *p* = 0.556) on the lamina propria area, there was a significant interaction (*F*_3_ = 4.0, *p* = 0.026), with a thicker lamina propria observed in fish fed without additives in the control diet conversely with additives in those fish fed the BSF50 and BSF75 diets (Figure 2).

**Figure 3:**
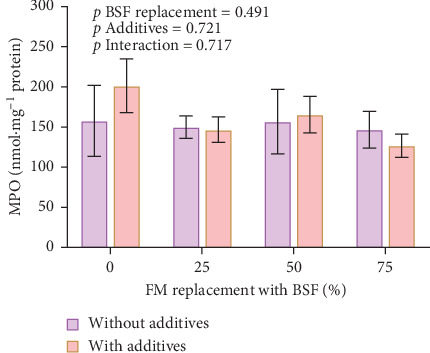
Myeloperoxidase of yellowtail kingfish hindgut sections. Values are in nmol.mg^−1^ protein ± SE (*n* = 3).

There was no significant effect of BSF replacement level (*F*_3_ = 0.84, *p* = 0.491), addition of additives (*F*_1_ = 0.13, *p* = 0.721) or interaction of these terms (*F*_3_ = 0.45, *p* = 0.717) on MPO concentration in the hindgut (Figure 3).

### 3.5. Blood Biochemistry

The level of BSF replacement significantly impacted serum cholesterol levels, with levels decreasing as BSF replacement increased (Table 7). Fish-fed BSF75 diet had significantly lower serum cholesterol (LSM 5.1 ± 0.3 mM) than all other treatments, while fish fed with BSF0 had significantly higher cholesterol (LSM 7.7 ± 0.3 mM) than all other treatments (*F*_3_ = 13.179, *p* < 0.001). There was no significant effect of additives nor an interaction between replacement level and additives on serum cholesterol levels following the Benjamini–Hochberg procedure.

Similarly, BSF replacement level had a significant effect on serum urea, with fish fed the BSF25 diet having the highest urea values (LSM 5.3 ± 0.3 mM), while those fed the BSF50 diet had the lowest urea values (LSM 3.7 ± 0.3 mM) (Table 7). There was no significant effect of dietary additives or interaction between BSF replacement level and additives on serum urea values. All other biochemical parameters showed no significant effects of BSF replacement, dietary additives, or their interaction (Table 7).

### 3.6. Gene Expression

There were no significant effects of BSF inclusion level, dietary additives, or their interaction on expression levels of any genes in the gut, liver, or brain (Tables 8–10).

### 3.7. Microbiome

Demultiplexed sequences yielded a total of 643,019 reads, with an average of 16,922 ± 1552 non-chimeric reads (~86.6%) inferred into 324 ASVs. After filtering for singletons and sequences assigned to mitochondria and chloroplasts, an average of 17.4 ± 9.8 ASVs per sample was obtained. After taxonomy-based filtering, the final dataset comprised 16 unique phyla, 86 orders, 146 families, and 215 genera. Each sample was normalised to an even depth of 1819 reads for diversity and compositional analysis. At this depth, bacterial diversity reached maximum saturation in all samples except the first four in the rarefaction plot (Figure S1). Phylogenetic data were further refined to rename ambiguous taxa such as “*Clostridium* sensu stricto 1-9” to “*Clostridium*” and group uncultured, unclassified, and ambiguous taxa (e.g., UBA12411) as “Unclassified.”

**Figure 4:**
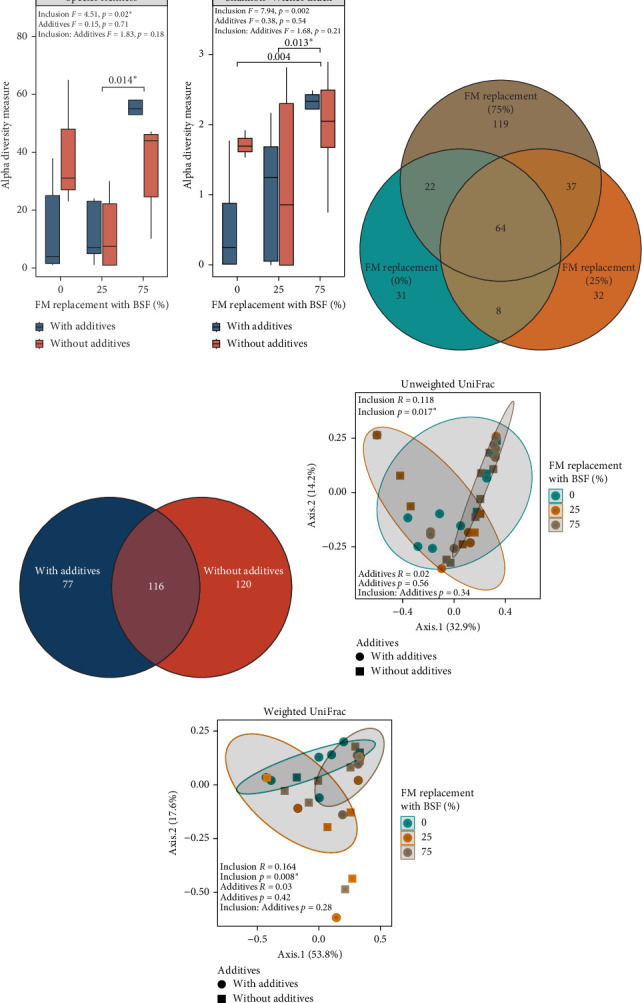
Alpha-beta diversity analysis of gut microbiota in yellowtail kingfish after a 33-day feeding trial using black soldier fly (BSF) larvae meal as a replacement for fishmeal at 25% and 75% with and without garlic and tuna hydrolysate additives. Diversity of gut bacteria in terms of (A) species richness and (B) Shannon–Weiner index. Number of shared and unique ASVs at different BSF inclusion levels (C) and with or without dietary additives (D). Beta-diversity PCoA plot based on unweighted (E) and weighted (F) UniFrac distance metric. *⁣*^*∗*^*p* < 0.05.

ASV richness was significantly affected by BSF replacement level, with greater richness in the BSF75 diets than in the BSF25 diets (Figure 4A). There were no significant effects of additives or the interaction of BSF replacement level and additives for species richness (Figure 4A). There was also a significant effect of BSF replacement levels on the Shannon diversity index, with greater diversity in BSF75 diets than in BSF0 and BSF25 diets (Figure 4B). There were no significant effects found for additives or the interaction of BSF replacement and additives for diversity index (Figure 4B). The number of unshared bacterial ASVs was higher in the BSF75 group (119) compared to the BSF25 group (32) and the BSF0 diet (31), with 64 ASVs shared among these three different diets (Figure 4C). More unique ASVs were observed in samples without additives (120) than those with additives (77) (Figure 4D). There was a significant effect of BSF replacement level on microbial community composition for both unweighted (*R*^2^ = 0.118, *p* = 0.017) and weighted (*R*^2^ = 0.164, *p* = 0.008) UniFrac distance metrics, but no significant effects of additives or interaction between BSF replacement level and additives (Figure 4E,F).

**Figure 5:**
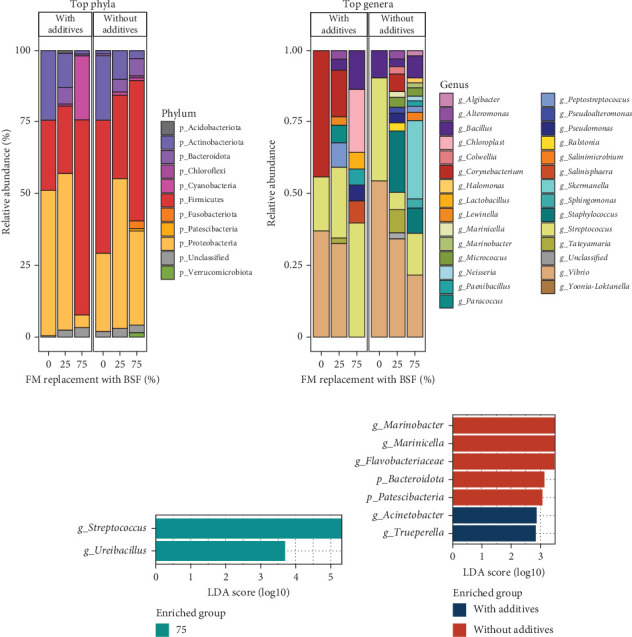
Gut bacterial composition in yellowtail kingfish after feeding trial. Relative abundance (RA) of bacteria at phylum (A) and genus (B) levels. Differential abundance (DA) of gut microbiota in yellowtail kingfish after feeding trial at different BSF replacement levels (C) and with or without dietary additives (D). A linear discriminant analysis (LDA) score of 2.0 and above and *p* < 0.05 was considered statistically significant. Phyla and genera with >1% abundance was considered for RA and DA analysis.

Proteobacteria-dominated bacterial communities were observed in fish in the BSF0 diets (57.7%) and BSF25 (71.9%) diets. Firmicutes comprised 31.3% and 18.8% of the composition in these two groups, respectively. By contrast, the abundance of Firmicutes was the most abundant phylum in the BSF75 dietary group, reaching 66.2%, along with 27% Proteobacteria. Together, these two phyla made up 90% of the composition in all samples, regardless of diet (Figure 5A). In addition to these two phyla, Actinobacteria accounted for 2%–10% abundance in all dietary groups (Figure 5A). In fish-fed diets without additives, Firmicutes were 8% more abundant (43.5%) compared to those with additives (35.8%). Conversely, Proteobacteria and Actinobacteria showed 5% higher abundance in fish-fed diets with additives compared to those without (Figure 5A).

At the genus level, *Vibrio* comprised about half of the read abundance in two dietary groups: BSF25 (58.3%) and BSF0 (56.8%). The second and third most abundant genera in these two groups were *Streptococcus* (23% in BSF0, 7.9% in BSF25) and *Corynebacterium* (6.8% in BSF0, 4.9% in BSF25) (Figure 5B). In the BSF75 group, the main genera were *Streptococcus* (38%), *Bacillus* (12.7%), and *Vibrio* (11.4%), with a significant percentage of other bacteria (21.3%) (Figure 5B). *Streptococcus* had a significantly higher abundance in fish from BSF75 diets compared to other groups (Figure 5C). Additionally, the genus *Acinetobacter* and the phylum Bacteroidota showed significantly higher abundance in dietary groups with and without additives, respectively (Figure 5D).

## 4. Discussion

This study demonstrated that the replacement of FM with BSF at levels ≤50% did not impact the performance of juvenile YTK. While our study observed reduced growth and feed intake at the highest inclusion level (75%, 300 g/kg), the findings at lower inclusion levels (25%–50%) are consistent with studies on Atlantic salmon (*S. salar*), Nile tilapia (*O. niloticus*), and rainbow trout (*O. mykiss*), where BSF meal has been shown to replace FM without negatively affecting growth performance or feed efficiency [12, 16, 24, 26, 27].

In the current study, however, there was a reduction in growth of the fish fed the diet in which 75% of FM was replaced. This decrease in growth performance was driven only by a reduction in feed intake and not by an impact on FCR. This suggests that a palatability issue, rather than a problem with digestibility. Studies with juvenile turbot (*S. maximus*) and rainbow trout (*O. mykiss*) have also found reduced growth linked to the palatability and feed intake of BSF meal [26, 28, 52, 53]. The inclusion of garlic at 1% and tuna hydrolysate at 3% in the diets did not improve the intake of the diets or increase the growth of the fish in this study suggesting their palatability enhancement properties were lacking at these inclusion levels. Some studies have shown that feeding BSF larvae on fish offal or seaweeds can improve the palatability of the meal to salmonids, suggesting that altering the substrate used to grow the BSF larvae may improve the palatability of the final manufactured diets [16, 54–56]. In this study, the BSF meal was produced using larvae grown on horticultural waste. This substrate is more readily available and can be consistently supplied to the local producer, resulting in a regular supply of BSF meal with the desired chemical and AA compositions.

The histological evaluation found that the fish in this study were in good health, and these findings align with previous research that has shown similar health outcomes in farmed fish when fed insect meal [57]. The liver showed adequate glycogen reserves, suggesting favourable energy storage and metabolic health [58]. The presence of lipid in small, dispersed droplets rather than in large vacuoles suggests that the fish are effectively metabolising and storing fats, which is a positive indicator of liver function and overall health [59]. Similarly, BSF diets showed no significant impact on the biochemical parameters associated with liver function. In the hindgut, the elongated villi with large enterocytes and a high number of mucous cells across all treatments suggest efficient digestive and absorptive capabilities. The presence of numerous absorptive droplets in the hindgut enterocytes further supports the notion of efficient nutrient absorption [60].

The minimal presence of extraneous cells, such as lymphocytes or cells undergoing diapedesis, and the typically thin lamina propria, suggest low levels of inflammation and a healthy gut environment across all BSF replacement diets. This is crucial as gut health directly impacts the overall health and growth performance of fish [61]. There was some evidence of an antagonistic interaction between additives and higher BSF replacement levels on the thickness of the lamina propria, whereby additives reduced the thickness of the lamina propria in fish-fed BSF0 diets but increased the thickness in fish-fed BSF50 and BSF75 diets. Nevertheless, all the gut morphology results fell within a healthy range for juvenile YTK, thus not raising any concerns about enteritis in these fish due to FM replacement [40, 62]. MPO results further support the finding of a healthy gut environment across all dietary treatments. MPO, a marker for neutrophil activity and thus an indicator of inflammation, showed no significant differences in levels across treatments, and this aligns with the minimal presence of inflammatory cells observed in gut histology. Together, these findings indicate that the dietary treatments maintained a low-inflammatory state in the gut, which is essential for sustaining overall health and growth performance. The absence of an inflammatory response, even with substantial replacement of FM with BSF meal, further underscores the potential of BSF meal as a viable alternative protein source in aquafeeds without compromising gut health [40, 63].

BSF meal replacement had a significant impact on blood cholesterol, with cholesterol significantly decreasing with an increase in BSF replacement and fish fed with the BSF75 diet having the lowest cholesterol levels. This reduction in serum cholesterol is likely due to the lower cholesterol content in diets with reduced FM [64]. In addition, the presence of chitin in BSF larvae may have contributed to this effect, as chitin has been shown to interfere with lipid absorption and promote cholesterol excretion in fish and other animals [28, 65]. It is important to note that while lower cholesterol levels were observed with increasing BSF inclusion, these levels did not reach the threshold for hypocholesterolemia, a condition that can increase susceptibility to bacterial infections, such as those caused by *Lactococcus garvieae*, as described in studies in *Seriola quinqueradiata* [66]. Should dietary cholesterol levels become a concern, particularly in cases where extreme FM replacement is used, this issue could be addressed through targeted supplementation of cholesterol or other lipids, thereby supporting the immune function of the fish [67].

BSF replacement also significantly impacted serum urea levels, with fish fed the BSF25 diet exhibiting the highest urea values, and those fed the BSF50 diet showing the lowest. This variation in urea levels suggests that BSF replacement influences nitrogen metabolism in fish. The observed highest urea levels in the BSF25 diet may indicate increased protein catabolism or altered nitrogen excretion pathways at this replacement level, while the lowest levels at the BSF50 diet could reflect optimal protein utilisation or metabolic balance [41, 60]. Interestingly, the differences in serum urea levels correlate with the variations in whole-body protein content and retention efficiencies. Fish fed the BSF50 diet had a significantly lower whole-body protein content compared to those fed the BSF75 diet (64.5% vs. 66.8%), which suggests that protein utilisation might differ across these diets. Despite these differences, protein retention across the treatments was not significantly different, indicating that the observed variations in urea levels may reflect changes in nitrogen metabolism rather than direct differences in protein retention efficiency. This could suggest that at the BSF25 replacement level, there is a potential for increased protein breakdown, while the BSF50 diet may offer a more balanced nitrogen metabolism, optimising protein utilisation. Additionally, the lack of significant differences in ADCs of protein across the diets supports the notion that differences in serum urea and whole-body protein content are more related to metabolic processes than to differences in dietary protein absorption.

There were no significant differences in gene expression in the gut, liver, and brain tissues of YTK-fed diets containing BSF or additives. The lack of significant differences in gene expression in the gut tissues suggests that BSF replacement and dietary additives do not substantially impact the gut's regulatory mechanisms involved in digestion, absorption, and gut health. This is in line with histological observations that indicated the gut structural integrity and function were maintained regardless of BSF replacement level [68]. The stable gene expression profile may reflect the gut's adaptive capacity to dietary changes without inducing stress or inflammation, as also suggested by the minimal presence of extraneous cells, low inflammation levels noted in histological analyses, and the lack of difference in MPO [69]. In the liver, the absence of significant changes in gene expression implies that BSF replacement and additives did not disrupt normal hepatic functions such as lipid metabolism, detoxification, and energy storage, as supported through the measured biochemical parameters relating to liver function The adequate glycogen reserves and lipid storage observed in the histological analyses supports this finding, suggesting that liver cells are not undergoing significant stress or metabolic disruption due to dietary changes [70]. For the brain tissues, the stable gene expression suggests that the regulation of appetite, metabolism, digestive processes, and growth of the fish are not adversely affected by BSF replacement or dietary additives and remain intact under these dietary conditions [71].

The microbiome analysis revealed significant differences in bacterial diversity and composition in the hindgut of the fish in response to varying levels of BSF replacement levels in the diets, highlighting the substantial impact of diet on gut microbial communities. The alpha diversity, as indicated by ASV richness and the Shannon diversity index, was significantly higher in the BSF75 inclusion group, and fish fed this diet also exhibited a greater number of unique ASVs. This suggests that the replacement of FM with higher levels of BSF promotes a richer and more diverse bacterial community. This result is similar to that found in Atlantic salmon, where BSF meal improved gut microbiome diversity and evenness with no adverse impacts on growth performance or gut histology [72]. The beta-ordination analysis further demonstrated that BSF replacement levels significantly affected the gut microbiota's composition. The distinct clustering observed in beta-diversity plots suggests that different replacement levels of BSF led to notable shifts in the microbial community structure. Similarly, BSF meal increased gut diversity and altered bacterial composition in rainbow trout (*O. mykiss*) [73].

At the phylum level, Proteobacteria and Firmicutes were dominant across all diet groups, but their RAs varied significantly with BSF replacement. The BSF75 group had a notably higher proportion of Firmicutes (66.2%) and a lower proportion of Proteobacteria (27%) compared to other groups. This shift in the dominant phyla may suggest a potential impact on gut health and metabolic functions, as Firmicutes are commonly associated with fermentation and energy extraction from the diet [74], although no direct evidence of such effects was observed in this study. At the genus level, this study identified significant shifts in the dominant genera between dietary treatments. In the BSF75 replacement group, *Streptococcus* (38%) and *Bacillus* (12.7%) were more prevalent, while *Vibrio* dominated in the BSF0 and BSF25 groups (23% and 7.9%, respectively). The increased presence of beneficial genera like *Bacillus* in the gut has been linked to enhanced gut health and pathogen resistance [75]. However, the higher abundance of *Streptococcus*, an opportunistic fish pathogen, suggests that increasing BSF replacement levels could potentially impact the immune responses and health outcomes of YTK. Despite these differences in gut microbiota, there was little evidence of changes in gut function or health with increasing BSF inclusion in the diet. Having both beneficial and detrimental bacteria in the gut creates a complex environment where the overall outcome depends on the balance between these microbial populations. If beneficial bacteria such as *Bacillus* can outcompete and suppress the growth of potentially harmful bacteria like *Streptococcus* species, the gut environment may remain healthy. However, if the detrimental bacteria dominate, it could lead to dysbiosis, impaired immune function, and increased susceptibility to diseases.

## 5. Conclusions

This study demonstrates that BSF larvae meal can partially replace FM in juvenile YTK diets without compromising growth or health. Replacement levels up to 50% supported normal growth and feed efficiency, while 75% inclusion reduced performance due to lower feed intake. Histological, biochemical, and gene expression results indicated maintained physiological health, and microbiome analysis showed increased diversity at higher BSF levels. These findings support the use of BSF meal as a sustainable alternative protein source in aquafeeds, with further work needed to improve palatability at high inclusion levels.

## Figures and Tables

**Table 1:** Proximate composition and amino acid profile of the partially defatted back soldier fly (BSF) larval meal compared to fishmeal (FM).

Nutrient composition	BSF	FM
Energy (kJ·100 g^−1^)	2080	1919
Ash residue (g·100 g^−1^)	3.9	20.7
Protein (g·100 g^−1^)	71.4	67.8
Fat (g·100 g^−1^)	23.1	6.1
Amino acids (g·100 g^−1^)		
Alanine	3.4	4.0
Arginine	2.4	3.9
Aspartic acid	6.2	5.9
Cystine	0.1	0.2
Glutamic acid	6.1	8.3
Glycine	2.5	4.2
Histidine	1.2	2.2
Isoleucine	2.3	2.7
Leucine	4.0	4.7
Lysine	4.3	5.1
Methionine	1.1	2.0
Phenylalanine	2.3	2.5
Proline	2.7	2.7
Serine	2.2	2.5
Taurine	0.0	0.5
Threonine	2.1	2.7
Tyrosine	3.1	2.2
Valine	3.4	3.3

**Table 2:** Formulation of the experimental diets and their target proximate compositions.

Ingredients (g·kg^−1^)	BSF0^a^	BSF0 + additives	BSF25	BSF25 + additives	BSF50	BSF50 + additives	BSF75	BSF75 + additives
Fishmeal	400	370	300	270	200	170	100	80
BSF meal	0	0	100	100	200	200	300	300
Poultry meal	70	70	70	70	70	70	70	70
Lupin kernel	100	100	100	100	100	100	100	100
Blood meal	70	80	79	90	86	97	97	100
Wheat flour	149	133	140	121	127	108	114	95
Meat meal	50	50	50	50	50	50	50	50
Tuna hydrolysate	0	30	0	30	0	30	0	30
Garlic powder	0	10	0	10	0	10	0	10
Fish oil	60	60	60	60	60	60	60	60
Poultry oil	60	57	50	47	40	37	30	27
Yttrium oxide	1	1	1	1	1	1	1	1
Cellulose	3	0	0	0	0	0	0	0
DL methionine	13	13	14	14	18	18	18	18
Taurine	10	10	10	10	10	10	10	10
Monosodium phosphate	5	5	10	9	15	14	20	18
Calcium carbonate	0	2	7	9	14	16	21	22
Vitamin premix	5	5	5	5	5	5	5	5
Stay C	1	1	1	1	1	1	1	1
Choline chloride	3	3	3	3	3	3	3	3

^a^Pilmer et al. [36].

**Table 3:** Genes analysed in hindgut, liver, and brain samples by RT-qPCR.

Class	Gene	Abbreviation	Tissue
Immune	Interleukin 1	*itl-1*	Liver
	Interleukin 8	*itl-8*	Liver
	Catalase	*cat*	Intestine
	Superoxide dismutase	*sod*	Liver, intestine
	Glutathione peroxidase 1	*gpx-1*	Intestine

Growth	Integumentary mucin	*i-mucin*	Intestine
	Mucin 2	*mucin-2*	Intestine
	Insulin-like growth factor 1	*igf-1*	Liver, brain
	Insulin-like growth factor 2	*igf-2*	Liver, brain
	Peptide YY	*yy*	Intestine, brain
	Cholecystokinin	*cck*	Intestine, brain
	Trypsin	*try*	Intestine
	Chymotrypsin	*chy*	Intestine
	Carboxypeptidase A	*cpa*	Intestine

**Table 4:** Proximate composition of test diets as dry matter (DM) basis and amino acid composition (g.kg^−1^ as is basis).

	BSF0	BSF0 + additives	BSF25	BSF25 + additives	BSF50	BSF50 + additives	BSF75	BSF75 + additives
Proximate composition (dry matter basis %)								
Crude protein	51.6	49.3	51.5	51.3	51.8	52.1	50.8	51.9
Total lipid	17.4	21.8	17.6	17.2	17.1	16.7	17.3	17.4
Ash	13.3	12.1	12.4	12.1	11.7	11.4	11.9	11.1
Carbohydrate	17.7	16.8	18.5	19.4	19.4	19.8	20.0	19.6
Cholesterol	1.6	1.4	1.5	1.6	1.3	1.3	1.1	1.1
Amino acid (g.kg^−1^ as is basis)								
Alanine	26.2	27.7	28.5	29.7	28.9	30.5	29.9	31.0
Arginine	23.1	23.8	22.2	22.1	21.2	24.8	23.6	24.0
Aspartic acid	38.4	40.1	41.8	43.7	42.1	45.0	41.4	43.1
Cystine	2.5	1.4	1.3	1.1	0.0	0.0	0.0	0.0
Glutamic acid	59.5	60.2	70.0	63.0	65.6	66.8	57.9	57.4
Glycine	28.2	28.4	26.3	26.8	26.7	28.9	27.8	29.9
Histidine	10.2	11.3	11.8	12.9	11.4	13.8	12.3	12.7
Isoleucine	13.2	13.4	13.8	13.9	13.8	13.9	13.6	14.0
Leucine	29.4	31.7	32.6	34.7	32.3	35.8	33.9	34.3
Lysine	29.5	30.6	31.3	32.2	30.0	32.6	29.7	30.1
Methionine	21.5	21.5	22.7	22.5	25.8	26.7	24.3	24.8
Phenylalanine	16.4	17.6	18.4	19.6	18.2	20.4	19.0	19.4
Proline	20.1	21.2	22.0	22.6	23.2	23.0	23.2	24.4
Serine	16.9	17.9	18.7	19.6	18.9	20.5	19.8	20.3
Taurine	11.7	11.2	11.1	10.1	10.9	10.5	9.8	10.5
Threonine	16.1	16.7	17.5	18.3	17.1	18.8	17.5	18.1
Tyrosine	12.0	13.6	15.0	15.2	16.0	17.4	17.9	18.5
Valine	19.3	20.8	21.5	23.3	22.1	24.2	24.3	24.6
Total amino acid	394.2	409.1	426.5	431.3	424.2	453.6	425.9	437.2

**Table 5:** Performance parameters of yellowtail kingfish for each treatment.

Performance parameter	BSF0	BSF0 + additives	BSF25	BSF25 + additives	BSF50	BSF50 + additives	BSF75	BSF75 + additives	Two-way ANOVA		
									*p* BSF replacement	*p* Additive	*p* Interaction
Initial body weight (g)	58.8 ± 0.2	58.7 ± 0.3	58.4 ± 0.1	58.6 ± 0.2	58.6 ± 0.1	58.7 ± 0.1	58.6 ± 0.2	58.6 ± 0.2	—	—	—
Final body weight (g)	204.5 ± 5.9	212.7 ± 3.2	209.0 ± 2.4	204.7 ± 2.7	200.8 ± 5.9	206.9 ± 1.6	190.7 ± 1.4	189.1 ± 1.1	**<0.001**	0.410	0.260
Weight gained (g.fish^−1^)	145.7 ± 5.8	154.0 ± 3.4	150.6 ± 2.4	146.0 ± 2.7	142.2 ± 5.9	148.1 ± 1.7	132.1 ± 1.3	130.5 ± 0.9	**<0.001**	0.424	0.253
Feed intake (g.fish^−1^)	152.4 ± 7.0	169.3 ± 1.7	157.8 ± 3.1	157.7 ± 3.5	152.4 ± 3.0	159.8 ± 2.2	147.1 ± 9.0	141.9 ± 2.8	**0.017**	0.169	0.142
SGR (%.day^−1^)	3.77 ± 0.08	3.90 ± 0.06	3.86 ± 0.04	3.79 ± 0.04	3.73 ± 0.09	3.82 ± 0.03	3.57 ± 0.02	3.55 ± 0.01	**<0.001**	0.459	0.240
FCR	1.07 ± 0.02	1.09 ± 0.03	1.04 ± 0.01	1.07 ± 0.01	1.05 ± 0.03	1.07 ± 0.02	1.09 ± 0.06	1.07 ± 0.02	0.783	0.555	0.793

*Note*: Values are mean ± SE (*n* = 3). *p*-Values in bold indicate significant differences (*p*  < 0.05).

Abbreviations: FCR, feed conversion ratio; SGR, specific growth rate.

**Table 6:** Whole-body proximate comparison, apparent digestibility, and retention efficiency of nutrients in yellowtail kingfish under different treatments (*n* = 3).

	Initial	Final										
		Without additives				With additives				ANOVA		
		BSF0	BSF25	BSF50	BSF75	BSF0	BSF25	BSF50	BSF75	*p* BSF replacement	*p* Additives	*p* Interaction
Proximate composition (%, DM)												
Crude protein	69.9 ± 0.3	64.5 ± 0.4	65.3 ± 0.1	63.8 ± 0.3	65.7 ± 0.9	66.5 ± 1.3	65.6 ± 0.5	65.3 ± 0.7	68.0 ± 0.5	**0.033**	**0.005**	0.517
Crude fat	12.9 ± 0.2	22.1 ± 0.3	23.8 ± 1.5	25.9 ± 0.3	22.8 ± 0.4	20.6 ± 1.2	23.6 ± 0.8	23.5 ± 1.4	22.2 ± 0.3	**0.013**	0.104	0.656
Crude ash	11.6 ± 0.1	9.8 ± 0.3	9.3 ± 0.1	9.1 ± 0.1	9.8 ± 0.1	9.7 ± 0.4	9.4 ± 0.2	9.5 ± 0.3	9.8 ± 0.1	0.089	0.653	0.611
Apparent digestibility (%)												
Protein	—	79.0 ± 1.4	79. ± 20.6	79.8 ± 1.1	79.8 ± 1.2	—	—	—	—	0.9 ± 29	—	—
Energy	—	76.8 ± 1.6	76.1 ± 0.4	76.9 ± 1.0	75.1 ± 0.6	—	—	—	—	0.618	—	—
Dry matter	—	84.1 ± 0.6	82.4 ± 0.9	83.7 ± 0.7	83.3 ± 0.9	—	—	—	—	0.479	—	—
Retention efficiency (%)												
Protein	—	31.8 ± 1.8	34.5 ± 0.2	33.1 ± 1.3	33.7 ± 1.1	35.8 ± 1.6	33.6 ± 0.2	33.7 ± 1.1	35.8 ± 0.6	0.674	0.096	0.215
Fat	—	37.7 ± 1.7	42.5 ± 3.0	48.3 ± 1.0	40.2 ± 2.2	28.2 ± 1.6	41.7 ± 1.8	43.8 ± 2.5	40.4 ± 0.3	**<0.001**	**0.017**	0.091
Energy	—	29.5 ± 1.5	31.7 ± 0.5	32.5 ± 0.9	30.0 ± 1.3	27.9 ± 0.5	30.5 ± 0.6	31.1 ± 0.5	30.6 ± 0.3	**0.014**	0.180	0.574

*Note*: Values are mean ± SE (*n* = 3). *p*-Values in bold indicate significant difference (*p* < 0.05).

**Table 7:** Biochemical analysis of serum collected from yellowtail kingfish after 33-day growth trial values are shown ± SE.

Additive inclusion	Diet	AST (U.L^−1^)	ALT (U.L^−1^)	GLDH (U.L^−1^)	Lipase (U.L^−1^)	Urea (m.mol.L^−1^)	Cholesterol (m.mol.L^−1^)	Trig (m.mol.L^−1^)	Total protein (g.L^−1^)
Without additives	BSF0	119.8 ± 25.6	8.2 ± 1.3	30.8 ± 6.8	9.0 ± 0.3	4.2 ± 0.4	8.3 ± 0.4	2.5 ± 0.2	41.3 ± 1.1
	BSF25	161.0 ± 105.7	10.5 ± 3.4	46.1 ± 6.1	14.5 ± 4.7	5.2 ± 0.4	7.0 ± 0.3	2.7 ± 0.3	43.0 ± 1.2
	BSF50	73.2 ± 10.8	7.3 ± 0.7	46.7 ± 7.7	13.8 ± 4.3	3.7 ± 0.2	6.4 ± 0.5	2.7 ± 0.3	40.5 ± 1.9
	BSF75	104.7 ± 39.1	8.5 ± 1.3	45.6 ± 8.2	13.8 ± 4.7	4.0 ± 0.5	5.5 ± 0.5	2.4 ± 0.3	39.3 ± 2.2

With additives	BSF0	61.3 ± 16.3	8.0 ± 2.0	47.8 ± 11.2	13.3 ± 4.1	5.0 ± 0.4	7.2 ± 0.6	2.3 ± 0.2	41.0 ± 1.1
	BSF25	102.0 ± 25.9	10.3 ± 1.7	36.2 ± 6.0	14.3 ± 4.3	5.5 ± 0.4	6.8 ± 0.3	2.3 ± 0.2	40.7 ± 1.3
	BSF50	181.5 ± 85.5	13.0 ± 3.3	40.6 ± 3.8	15.0 ± 4.3	3.8 ± 0.4	5.8 ± 0.3	2.4 ± 0.2	40.8 ± 1.2
	BSF75	121.8 ± 36.3	11.7 ± 2.0	54.9 ± 3.1	13.2 ± 4.2	4.0 ± 0.2	4.8 ± 0.4	2.0 ± 0.2	40.2 ± 1.1

*p* BSF replacement		0.870	0.685	0.437	0.838	**<0.001**	**<0.001**	0.600	0.539

*p* Additives		0.958	0.171	0.608	0.689	0.284	0.041	0.063	0.717

*p* Interaction		0.363	0.468	0.197	0.928	0.709	0.728	0.967	0.712

*Note*: *p*-Values were calculated using two-way ANOVA with bold values indicating a significant difference with the Benjamini–Hochberg procedure (*n* = 3).

**Table 8:** Gene expression of chymotrypsin (*chy*), trypsin (*try*), carboxypeptidase A (*cpa*), cholecystokinin (*cck*), peptide YY (*yy*), mucin 2 (*mucin-2*), integumentary mucin (*i-mucin*), glutathione peroxidase 1 (*gpx-1*), catalase (*cat*), and superoxide dismutase (*sod*) in the hindgut of yellowtail kingfish after 33-day growth trial.

Additive inclusion	Diet	Digestive enzyme and hormone-encoded genes					Immune-related genes		Oxidative stress-related genes		
		*chy*	*try*	*cpa*	*cck*	*yy*	*mucin-2*	*i-mucin*	*gpx-1*	*cat*	*sod*
Without additives	BSF0	2.6 ± 0.1	3.8 ± 0.1	2.3 ± 0.3	4.2 ± 0.1	3.1 ± 0.1	4.2 ± 0.1	3.7 ± 0.1	3.7 ± 0.2	3.3 ± 0.3	4.0 ± 0.1
	BSF25	2.6 ± 0.1	3.7 ± 0.1	2.1 ± 0.2	4.2 ± 0.1	3.7 ± 0.1	4.2 ± 0.1	3.9 ± 0.1	3.7 ± 0.1	3.8 ± 0.3	3.9 ± 0.1
	BSF75	2.6 ± 0.1	3.7 ± 0.1	2.2 ± 0.2	4.3 ± 0.1	3.3 ± 0.5	4.2 ± 0.0	4.0 ± 0.0	3.9 ± 0.0	3.9 ± 0.2	4.0 ± 0.0

With additives	BSF0	2.9 ± 0.2	3.6 ± 0.2	2.4 ± 0.1	4.3 ± 0.1	3.5 ± 0.2	4.2 ± 0.1	3.8 ± 0.2	3.8 ± 0.0	3.9 ± 0.0	4.0 ± 0.1
	BSF25	2.4 ± 0.1	3.7 ± 0.0	1.5 ± 0.2	4.4 ± 0.1	3.6 ± 0.3	4.3 ± 0.1	4.0 ± 0.1	4.0 ± 0.1	3.9 ± 0.2	4.0 ± 0.1
	BSF75	2.8 ± 0.1	3.9 ± 0.1	2.4 ± 0.5	4.4 ± 0.1	3.5 ± 0.2	4.2 ± 0.1	4.0 ± 0.1	3.9 ± 0.2	4.0 ± 0.1	4.0 ± 0.0

*p* BSF replacement		0.164	0.505	0.141	0.192	0.492	0.973	0.195	0.569	0.312	0.657

*p* Additives		0.575	0.724	0.703	0.094	0.519	0.302	0.551	0.188	0.177	0.338

*p* Interaction		0.162	0.217	0.418	0.738	0.637	0.940	0.821	0.668	0.448	0.327

*Note*: *p*-Values calculated using two-way ANOVA (*n* = 3). Values are in mean relative expression level ±SE.

**Table 9:** Gene expression of interleukin 1 (*itl-1*), interleukin 8 (*itl-8*), insulin-like growth factor 1 (*igf-1*), insulin-like growth factor 2 (*igf-2*), and superoxide dismutase (*sod*) in the liver of yellowtail kingfish after 33-day growth trial.

Additive inclusion	Diet	Liver gene expression				
		*itl-1*	*itl-2*	*igf-1*	*igf-2*	*sod*
Without additives	BSF0	4.0 ± 0.1	3.1 ± 0.4	4.2 ± 0.1	4.1 ± 0.0	4.2 ± 0.0
	BSF25	3.9 ± 0.2	3.2 ± 0.4	4.2 ± 0.1	4.1 ± 0.1	4.1 ± 0.1
	BSF75	3.8 ± 0.2	2.9 ± 0.2	4.1 ± 0.0	4.1 ± 0.1	4.0 ± 0.1

With additives	BSF0	4.1 ± 0.2	3.1 ± 0.0	4.1 ± 0.1	4.0 ± 0.1	4.1 ± 0.1
	BSF25	3.9 ± 0.2	2.8 ± 0.3	4.2 ± 0.1	4.1 ± 0.1	4.0 ± 0.1
	BSF75	3.9 ± 0.1	2.8 ± 0.4	4.2 ± 0.0	4.1 ± 0.0	4.0 ± 0.0

*p* BSF replacement	—	0.485	0.720	0.513	0.781	0.182

*p* Additives	—	0.803	0.461	0.385	0.594	0.825

*p* Interaction	—	0.952	0.856	0.324	0.820	0.818

*Note*: Values are in mean relative expression level ±SE. *p*-Values calculated using two-way ANOVA (*n* = 3).

**Table 10:** Gene expression of peptide YY (*yy*), cholecystokinin (*cck*), insulin-like growth factor 1 (*igf-1*), and insulin-like growth factor 2 (*igf-2*) in the brain of yellowtail kingfish after a 33-day growth trial.

Additive inclusion	Diet	Brain gene expression			
		*yy*	*cck*	*igf-1*	*igf-2*
Without additives	BSF0	4.0 ± 0.1	4.2 ± 0.1	2.3 ± 0.1	3.5 ± 0.2
	BSF25	3.7 ± 0.1	4.2 ± 0.1	2.7 ± 0.2	3.5 ± 0.2
	BSF75	3.7 ± 0.2	4.2 ± 0.1	2.6 ± 0.2	3.7 ± 0.1

With additives	BSF0	3.9 ± 0.0	4.2 ± 0.1	2.9 ± 0.3	3.7 ± 0.2
	BSF25	3.7 ± 0.1	4.2 ± 0.1	2.4 ± 0.1	3.6 ± 0.1
	BSF75	3.7 ± 0.2	4.3 ± 0.1	2.6 ± 0.1	3.8 ± 0.0

*p* BSF replacement	—	0.331	0.478	0.983	0.296

*p* Additives	—	0.940	0.627	0.504	0.222

*p* Interaction	—	0.877	0.946	0.075	0.945

*Note*: Values are in mean relative expression level ±SE. *p*-Values calculated using two-way ANOVA (*n* = 3).

## Data Availability

The data that support the findings of this study are available from the corresponding author upon reasonable request.

## Supporting Information

Supporting Information Table S1. List of target genes analysed in *Seriola lalandi* (yellowtail kingfish), including their corresponding oligonucleotide (oligo) primer names and the specific tissues where gene expression was assessed. These genes include those involved in digestion, immune response, antioxidant defence, and growth regulation. Table S2. Details of qPCR primers and probes used for gene expression analysis in *Seriola lalandi*. This table provides the gene names, forward (F) and reverse (R) primer sequences, and corresponding probe numbers. Figure S1. Rarefaction plot showing the depth and saturation level of 38 samples. The red line indicates the lowest depth (1819) at which samples were normalised for further diversity and composition analysis.
