# Supplementary material for: Sustainable Fishmeal Alternatives: Impact of Partially Defatted Black Soldier Fly (Hermetia illucens) Meal on Growth and Health of Yellowtail Kingfish (Seriola lalandi)
Source: Aquac Nutr. 2025 Jun 19;2025:1804215. doi: 10.1155/anu/1804215 (PMC12202081; doi:10.1155/anu/1804215)
Supplement: Supporting Information — Table S1. List of target genes analysed in Seriola lalandi (yellowtail kingfish), including their corresponding oligonucleotide (oligo) primer names and the specific tissues where gene expression was assessed. These genes include those involved in digestion, immune response, antioxidant defence, and growth regulation. Table S2. Details of qPCR primers and probes used for gene expression analysis in Seriola lalandi. This table provides the gene names, forward (F) and reverse (R) primer sequences, and corresponding probe numbers. Figure S1. Rarefaction plot showing the depth and saturation level of 38 samples. The red line indicates the lowest depth (1819) at which samples were normalised for further diversity and composition analysis. [file 1804215.f1.docx]

Supplementary data

Supplementary Table 1. List of target genes analyzed in *Seriola lalandi* (Yellowtail Kingfish), including their corresponding oligonucleotide (oligo) primer names and the specific tissues where gene expression was assessed. The genes of interest include those involved in digestion (e.g., *trypsin, chymotrypsin, carboxypeptidase A, cholecystokinin*), immune response (e.g., *interleukin 8, mucin 2, i-mucin*), antioxidant defense (e.g., *catalase, superoxide dismutase, glutathione peroxidase 1*), and growth regulation (e.g., *insulin-like growth factor 1 and 2, peptide YY*).

| Gene name | Oligo Name | Tissue |
| --- | --- | --- |
| trypsin (try) | qSer-lal_trypsin-84-F | intestine |
| trypsin | qSer-lal-trypsin-84-R | intestine |
| chymotrypsin (chy) | qSer_lal_ chymotrypsin_40-F | intestine |
| chymotrypsin | qSer-lal_chymotrypsin-40-R | intestine |
| Carboxypeptidase A (cpa) | qSer-lal_carbo_27_F | intestine |
| carboxypeptidase | qSer-lal_carbo_27_R | intestine |
| cholecystokinin (cck) | qSer-lal_cck_2_F | intestine and brain |
| cholecystokinin | qSer-lal_cck_2_R | intestine and brain |
| interleukin 8 (itl8) | qSer_lal_ il8_62-F | liver |
| interleukin 8 | qSer-lal_il8-62-R | liver |
| i- mucin (i-muc) | qSer-lal_imuc_157_F | intestine |
| i- mucin | qSer-lal_imuc_157_R | intestine |
| mucin 2 (muc2) | qSer-lal_muc2_1_F | intestine |
| mucin 2 | qSer-lal_muc2_1_R | intestine |
| catalase (cat) | qSer-lal_cat_84_F | liver and intestine |
| catalase | qSer-lal_cat_84_R | liver and intestine |
| superoxide dismutase (sod) | qSer-lal_sod_84_F | liver and intestine |
| Superoxide dismutase | qSer-lal_sod_84_R | liver and intestine |
| glutathione peroxidase 1 (gpx1) | qSer-lal_GPX_2_F | liver and intestine |
| glutathione Peroxidase 1 | qSer-lal_GPX_2_R | liver and intestine |
| insulin-like growth factor 1 (igf1) | qSer-lal_igf1_1_F | liver and brain |
| insulin-like growth factor 1 | qSer-lal_igf1_1_R | liver and brain |
| insulin-like growth factor 2 (igf2) | qSer-lal_igf2_84_F | liver and brain |
| insulin-like growth factor 2 | qSer-lal_igf2_84_R | liver and brain |
| peptite YY | qSer-lal_yy_54_F | intestine and brain |
| peptite YY | qSer-lal_yy_54_R | intestine and brain |

Supplementary Table 2. Details of qPCR primers and probes used for gene expression analysis in *Seriola lalandi* (Yellowtail Kingfish). The table includes the gene names, forward (F) and reverse (R) primer sequences, and corresponding probe numbers. These primers target key genes involved in various physiological functions, including housekeeping genes (*elongation factor 1-α, 18S, β-actin*), immune response (*interleukin 1, interleukin 8, mucin 2, i-mucin*), antioxidant defense (*catalase, superoxide dismutase, glutathione peroxidase 1*), growth regulation (*insulin-like growth factor 1 and 2, peptide YY*), and digestion (*trypsin, chymotrypsin, carboxypeptidase A, cholecystokinin*).

| Gene name | Sequences | Probe’s number |
| --- | --- | --- |
| elongation factor 1-α -F | ggatggcatggagacaacat | 54 |
| elongation factor 1-α- R | atcttccatcccttgaacca | 54 |
| 18S-F | aggactccggttctattttgtg | 57 |
| 18S-R | cggccgtccctcttaatc | 57 |
| β-actin - F | cctgtcctgctcacagagg | 7 |
| β-actin - R | tgttgaaggtctcgaacatga | 7 |
| interleukin 1 (itl1) -F | gccaaacgctacagaacctt | 42 |
| interleukin 1 (itl1) -R | ccactttgggtgtcttggac | 42 |
| interleukin 8 (itl8) - F | tgactgagaggcatctctgattat | 62 |
| interleukin 8 (itl8) - R | ttccatttacttccttgatctgtg | 62 |
| i- mucin (i-muc) - F | tgtgctcctggttcgactc | 157 |
| i- mucin (i-muc) - R | acggtgcaggagtacttgaaa | 157 |
| mucin 2 (muc2) - F | cacctgtgaccagatgttgc | 1 |
| mucin 2 (muc 2) - R | gttcagggtcaatcagtttgg | 1 |
| catalase (cat) - F | gactgatcaaggaataaagaatctgtc | 84 |
| catalase (cat) - R | ttgcataatctgggttggtg | 84 |
| superoxide dismutase (sod)- F | gctccttccagaaaatgaaagag | 84 |
| superoxide dismutase (sod) - R | tccactctgcttgtcatagcc | 84 |
| glutathione peroxidase 1 (gpx1) -F | cacgggccttaccataatctt | 2 |
| glutathione peroxidase 1 (gpx1) -R | tccaggacggacatacttca | 2 |
| insulin-like growth factor 1 (igf1)-F | ttattatacctccatgtgtgagctg | 1 |
| insulin-like growth factor 1 (igf1)-R | tgaagcccagatccgttc | 1 |
| insulin-like growth factor 2 (igf2)-F | ccactcactttgccacacc | 84 |
| insulin-like growth factor 2 (igf2)-R | ctggacgaagacattttcctg | 84 |
| peptite YY -F | gcagagctcctgtttggtg | 54 |
| peptite YY-R | caccacatgtaggaatcgtca | 54 |
| cholecystokinin (cck)- F | gacatagtggccatatacaacagc | 2 |
| cholecystokinin-R | gcgttcgttcgctttctc | 2 |
| trypsin (tryp)- F | agctctgctgtcccattcc | 84 |
| trypsin-R | tgccacaactggagtgtcat | 84 |
| chymotrypsin (chy) -F | gctcccagtactcccaacaa | 40 |
| chymotrypsin_R | acactgctcattggacagca | 40 |
| carboxypeptidase A (cpa) -F | gcgtgacactggtcgttatg | 27 |
| carboxypeptidase -R | tcacatgtgggcttgatctg | 27 |

#
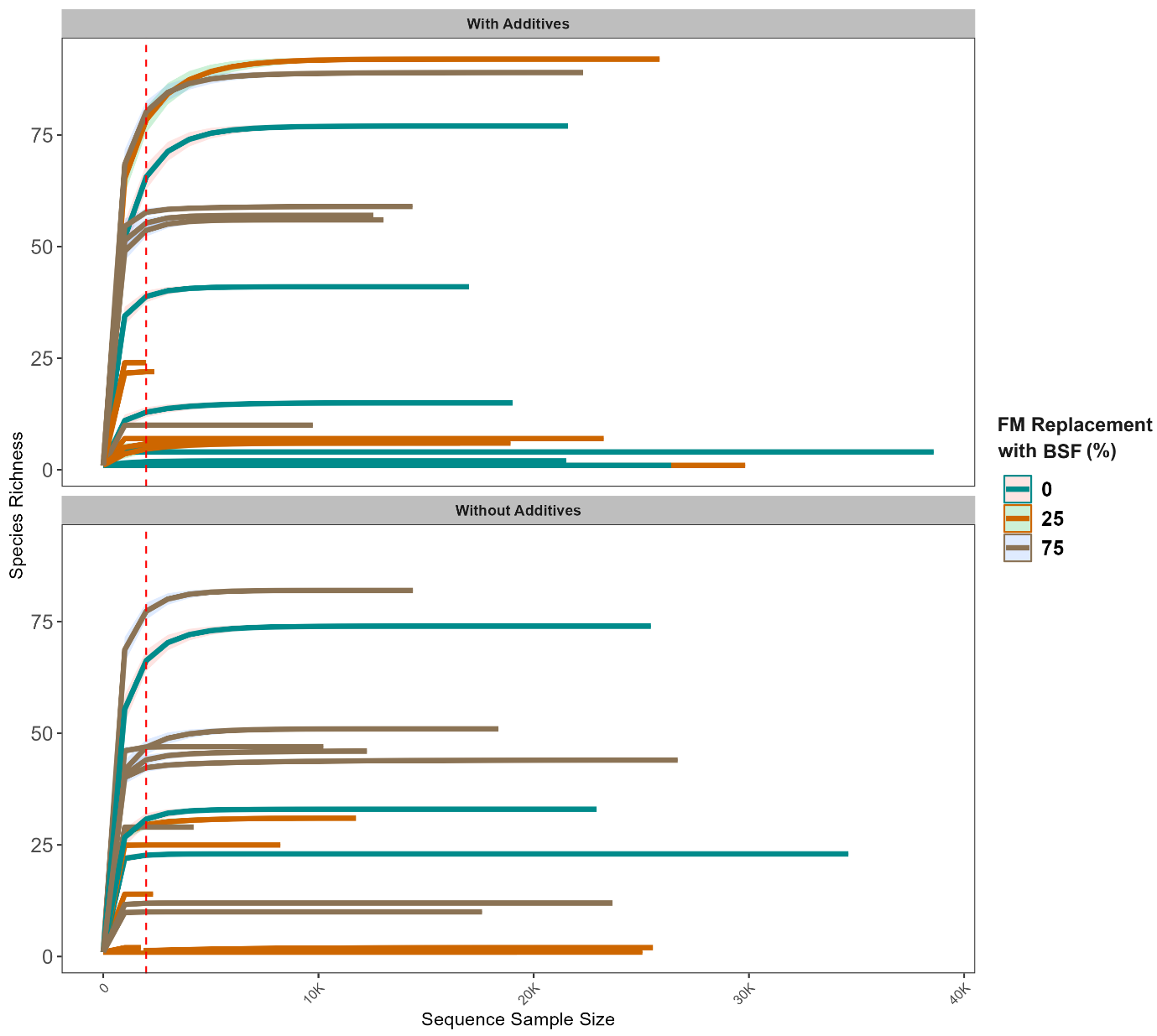


Supplementary Figure 1. Rarefaction plot showing the depth and saturation level of 38 samples. The red line indicates the lowest depth (1,819) at which samples were normalized for further diversity and composition analysis.
